# Supplementary material for: Topological edge states of interacting photon pairs emulated in a topolectrical circuit
Source: Nat Commun. 2020 Mar 18;11:1436. doi: 10.1038/s41467-020-14994-7 (PMC7080762; doi:10.1038/s41467-020-14994-7)
Supplement: Supplementary file 1 — Supplementary Information [file 41467_2020_14994_MOESM1_ESM.pdf]

Supplementary Information

Topological edge states of interacting photon pairs emulated in a topolectrical circuit

Olekhno *et al.*

Contents

Supplementary Note 1 – Analysis of the strong interaction limit $U \gg J$: Su-Schrieffer-Heeger model for doublons 3

Supplementary Note 2 – Eigenmode tomography 5

Supplementary Note 3 – Mapping of tight-binding model onto the topolectrical circuit: ideal lossless case 8

Supplementary Note 4 – Analysis of topolectrical circuit with realistic losses 12

Supplementary Note 5 – Distribution of elements in the experimental setup 16

Supplementary Note 6 – Mutual inductance 17

Supplementary Note 7 – Circuit impedance and doublon spectroscopy 19

Supplementary Note 8 – Scattering states 22

Supplementary Note 9 – Effects of disorder 24

Supplementary Note 10 – Evaluation of topological invariant from experimental data 25

Supplementary Note 1 – Analysis of the strong interaction limit $U \gg J$: Su-Schrieffer-Heeger model for doublons

To provide a simple proof of the topological origin of our model, we analyze the strong interaction limit, when $U \gg J$. In such a situation, doublon bands are well-separated from the continuum of two-photon scattering states so that the mixing between doublons and scattering states is negligible. As such, we introduce an effective doublon Hamiltonian which captures the dynamics of doublons excluding other redundant degrees of freedom.

To derive the effective doublon Hamiltonian, we start from the eigenvalue equations provided in the article main text (Methods section):

$$(\varepsilon - 2U)\beta_{2m,2m} = -2J\,\left[\beta_{2m+1,2m} + \beta_{2m,2m-1}\right] + P\beta_{2m-1,2m-1}\,, \tag{1}$$

$$(\varepsilon - 2U)\beta_{2m+1,2m+1} = -2J\,\left[\beta_{2m+2,2m+1} + \beta_{2m+1,2m}\right] + P\beta_{2m+2,2m+2}\,, \tag{2}$$

$$\varepsilon\beta_{m,n} = -J\,\left[\beta_{m+1,n} + \beta_{m-1,n} + \beta_{m,n+1} + \beta_{m,n-1}\right]\,,\ \ (m \neq n) \tag{3}$$

In the limiting case $U \gg J$, β_{mm} are the dominant coefficients of the doublon wave function with the rest of coefficients $\beta_{m+s,m}$ decaying with the index s. Therefore, we neglect all terms proportional to $\beta_{m+2,m}$, $\beta_{m+3,m}$, etc. in Supplementary Equations (1)-(3), treating terms proportional to $\beta_{m+1,m}$ as a perturbation.

Using the approximation $\beta_{m+1,m} \approx -\left(\beta_{mm} + \beta_{m+1,m+1}\right)/(2\,U)$, we derive the following approximate eigenvalue equation for doublon bands:

$$(\varepsilon - 2U - 2j)\beta_{2m,2m} = (j + P)\beta_{2m-1,2m-1} + j\,\beta_{2m+1,2m+1}\,, \tag{4}$$

$$(\varepsilon - 2U - 2j)\beta_{2m+1,2m+1} = j\,\beta_{2m,2m} + (j + P)\,\beta_{2m+2,2m+2}\,. \tag{5}$$

The equations for β_{11} and β_{NN} corresponding to the physical edges of the array are modified if compared

to the bulk sites:

$$(\varepsilon - 2U - j)\beta_{11} = (j + P)\,\beta_{22}\;, \tag{6}$$

$$(\varepsilon - 2U - j)\beta_{NN} = j\,\beta_{N-1,N-1}\;, \tag{7}$$

where $j = J^2/U$ is the effective doublon hopping rate associated with two consecutive single-particle tunnelings to the neighboring cavity. Here, the array length N is assumed to be odd.

The set of Supplementary Equations (4)-(5) corresponds to the Su-Schrieffer-Heeger (SSH) model [1] with two alternating tunneling amplitudes j and $j + P$, which is known to be the simplest one-dimensional topological model. The only difference from the canonical SSH model is the interaction-induced detuning of the edge sites by j captured by Supplementary Equations (6), (7).

Solving the system of Supplementary Equations (4)-(5) together with boundary conditions Supplementary Equations (6)-(7), we find two states with the localization ratio $z = \beta_{mm}/\beta_{m-2,m-2}$ given by

$$z_{1,2} = \frac{j+P}{2\,j^3}\left[2j\,P + P^2 \pm \sqrt{(2jP + P^2)^2 + 4\,j^4}\right]\;. \tag{8}$$

Edge-localized states correspond to $|z| < 1$. The energies of these states read:

$$\varepsilon_{1,2} = 2U + j - \frac{1}{2j}\left[2jP + P^2 \pm \sqrt{(2jP + P^2)^2 + 4j^4}\right]\;. \tag{9}$$

Supplementary Equation (8) shows that the higher-energy edge state ε_2 is localized for any $P \neq 0$, while the lower-energy state ε_1 disappears when

$$P > 0 \text{ or } P < -2J^2/U\;. \tag{10}$$

In our case with $U/J = 7.09$ and $P/J = -4.18$ the latter condition is fulfilled, which means that only the higher-energy state ε_2 persists. Note also that the condition Supplementary Equation (10) is equivalent to $|j + P| > j$ which ensures that the site (N, N) is the weak link edge and the respective edge state can be interpreted as the topological state inherent to SSH model.

Supplementary Note 2 – Eigenmode tomography

In an experimental situation, we can examine the excitation of the system applying voltage to various sites. However, we have no direct access to the system eigenmodes and associated probability distributions, which eventually limits our possibilities to observe topological physics in the designed setup.

To overcome this limitation, we elaborate on the procedure of eigenmode reconstruction (tomography) following the proposals of our recent work [2]. The key idea of this method is to collect the information on system excitation when the voltage is applied to various pairs of sites (m, n) symmetrically with respect to the diagonal. The eigenmode profile is then recovered as a sum of squares of voltages in all nodes of the circuit plotted as a function of the feeding point (m, n). In this section, we provide a summary of the proposed technique highlighting further applications of the developed method and its applicability to a wide class of systems described by tight-binding equations.

We consider a two-dimensional system depicted in Supplementary Figure 1. We describe the behavior of the system under external coherent driving with the following semi-empirical coupled mode equations:

$$\omega\, c_{mn} = \sum_{m',n'} H_{mn,m'n'}\, c_{m'n'} - i\gamma\, c_{mn} - i\kappa\, E_{mn}\ . \tag{11}$$

Here $H_{mn,m'n'}$ is the Hamiltonian of a closed system which is assumed Hermitian, γ quantifies the dissipation rate, and E_{mn} stands for the applied electromotive force (or injected current, depending on the interpretation of c_{mn} coefficients) at frequency ω. In an actual experimental situation, we can apply different drivings E_{mn} at different frequencies and measure the resultant intensity distribution $|c_{mn}|^2$. Our goal is to elaborate a protocol to reconstruct the profile of the system eigenmode $\beta_{mn}^{(k)}$ corresponding to the eigenfrequency ω_k which is the solution of the eigenvalue problem

$$\omega_k\, \beta_{mn}^{(k)} = \sum_{m',n'} H_{mn,m'n'}\, \beta_{m'n'}^{(k)}\ . \tag{12}$$

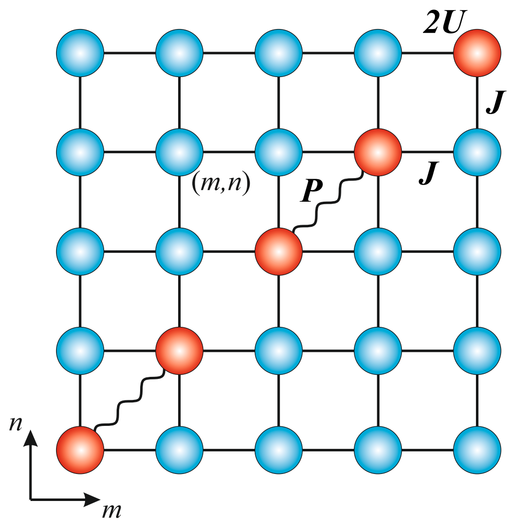

Supplementary Figure 1 Illustration of a two-dimensional setup used for the procedure of eigenmode tomography. For this technique, specific type of tight-binding model is not essential.

Since the Hamiltonian of the closed system is Hermitian, the eigenfrequencies ω_k are purely real and the eigenmodes $\beta_{mn}^{(k)}$ are mutually orthogonal, i.e.

$$\sum_{m,n} \beta_{mn}^{(k)} \left[\beta_{mn}^{(k')}\right]^* = \delta_{kk'} \,. \tag{13}$$

Furthermore, the modes $\beta_{mn}^{(k)}$ form a complete basis and the coefficients c_{mn} can be expanded as

$$c_{mn} = \sum_k \alpha_k \, \beta_{mn}^{(k)} \,. \tag{14}$$

Putting the expansion Supplementary Equation (14) into Supplementary Equation (11), we obtain:

$$\omega \sum_k \alpha_k \, \beta_{mn}^{(k)} = \sum_{m',n',k} H_{mn,m'n'} \, \alpha_k \, \beta_{m'n'}^{(k)} - i\gamma \sum_k \alpha_k \, \beta_{mn}^{(k)} - i\kappa \, E_{mn} = \tag{15}$$

$$\overset{(12)}{=} \sum_k \alpha_k \left(\omega_k - i\gamma\right) \beta_{mn}^{(k)} - i\kappa \, E_{mn} \,. \tag{16}$$

Using the orthogonality property Supplementary Equation (13), we recover that

$$\left(\omega - \omega_k + i\,\gamma\right) \, \alpha_k = -i\kappa \sum_{m,n} E_{mn} \left[\beta_{mn}^{(k)}\right]^* \,. \tag{17}$$

Physically, Supplementary Equation (17) expresses an intuitive fact that the larger the overlap of the driving profile with the eigenmode, the larger is the contribution of the eigenmode into the driven-dissipative system stationary state. On the other hand, the closer is the driving frequency to the resonance, the larger is the contribution of a given eigenmode to the driven-dissipative system stationary state. The coefficients c_{mn} are expressed as follows:

$$c_{mn} \overset{(14)}{=} \sum_k \alpha_k \, \beta^{(k)}_{mn} = -i\kappa \sum_{m',n',k} E_{m'n'} \, \frac{\left[\beta^{(k)}_{m'n'}\right]^* \, \beta^{(k)}_{mn}}{\omega - \omega_k + i\gamma} \,. \tag{18}$$

At this point we assume that the driving profile (a) includes only one or two points; (b) is symmetric with respect to the diagonal $m = n$. In the other words,

$$E_{mn} = \frac{E_0}{2} \, \left[\delta_{mp} \, \delta_{nq} + \delta_{mq} \, \delta_{np}\right] \,, \tag{19}$$

where p and q provide the coordinates of the excitation point(s). This assumption immediately simplifies the sum. Since the system is symmetric with respect to the diagonal, all non-degenerate eigenmodes are either even or odd; degenerate modes can also be enforced to satisfy this condition by choosing suitable linear combinations. Next, all odd modes have zero overlaps with symmetric pumping profile and they drop out of the sum. The remaining even modes have $\beta^{(k)}_{pq} = \beta^{(k)}_{qp}$. Hence,

$$c_{mn} = -i\kappa \, E_0 \sum_k \frac{\beta^{(k)}_{mn} \, \left[\beta^{(k)}_{pq}\right]^*}{\omega - \omega_k + i\gamma} \,. \tag{20}$$

Next, we construct the following quantity:

$$\begin{aligned} \mathfrak{I}(p,q) &= \sum_{m,n} |c_{mn}|^2 = \kappa^2 \, |E_0|^2 \sum_{m,n,k,k'} \frac{\beta^{(k)}_{mn} \, \left[\beta^{(k')}_{mn}\right]^* \, \left[\beta^{(k)}_{pq}\right]^* \, \beta^{(k')}_{pq}}{(\omega - \omega_k + i\gamma) \, (\omega - \omega_{k'} - i\gamma)} \\ &\overset{(13)}{=} \kappa^2 \, |E_0|^2 \sum_{k,k'} \frac{\delta_{kk'} \, \left[\beta^{(k)}_{pq}\right]^* \, \beta^{(k')}_{pq}}{(\omega - \omega_k + i\gamma) \, (\omega - \omega'_k - i\gamma)} = \kappa^2 \, |E_0|^2 \sum_k \frac{|\beta^{(k)}_{pq}|^2}{(\omega - \omega_k)^2 + \gamma^2} \,. \end{aligned} \tag{21}$$

Thus, for symmetric pumping into (p, q) and (q, p) with the same amplitudes $E_0/2$ and $E_0/2$ and equal phases the sum of squares of field amplitudes in all sites of the system reads:

$$\mathfrak{I}(p,q) \equiv \sum_{m,n} |c_{mn}|^2 = \kappa^2 \, |E_0|^2 \sum_k \frac{|\beta^{(k)}_{pq}|^2}{(\omega - \omega_k)^2 + \gamma^2} \,, \tag{22}$$

where the sum extends only over the symmetric eigenmodes. If the driving frequency is close enough to the eigenfrequency ω_k, the obtained distribution $\mathfrak{I}(p,q)$ will closely resemble the distribution $|\beta_{pq}^{(k)}|^2$, which is actually the eigenmode intensity (or two-photon probability distribution in the original 1D two-particle problem).

The outlined eigenmode reconstruction protocol works especially well once the spectral distance from the given mode to the rest of the modes exceeds the dissipation rate γ. In such a case, reconstruction of the eigenmode will be very precise as can be seen from the comparison made in the main text. This technique is also useful for the group of modes, e.g. doublon band, well-separated from the rest of the modes.

Note also that the developed eigenmode reconstruction technique is very general and applicable to a wide range of physical systems. In this work, we apply this protocol to LC circuits. Further identification of driven-dissipative model Supplementary Equation (11) with Kirchhoff's equations for the electric circuit is provided in Supplementary Notes 3-4.

Supplementary Note 3 – Mapping of tight-binding model onto the topolectrical circuit: ideal lossless case

As indicated in the article main text, the two-particle one-dimensional quantum problem with extended Bose-Hubbard Hamiltonian is described by the following system of tight-binding equations:

$$(\varepsilon - 2U)\ \beta_{2m+1,2m+1} = -2J\,\beta_{2m,2m+1} - 2J\,\beta_{2m+1,2m+2} + P\,\beta_{2m+2,2m+2}\ , \tag{23}$$

$$(\varepsilon - 2U)\ \beta_{2m,2m} = -2J\,\beta_{2m-1,2m} - 2J\,\beta_{2m,2m+1} + P\,\beta_{2m-1,2m-1}\ , \tag{24}$$

$$\varepsilon\,\beta_{mn} = -J\,\beta_{m-1,n} - J\,\beta_{m+1,n} - J\,\beta_{m,n-1} - J\,\beta_{m,n+1}\ \ (m \neq n)\ . \tag{25}$$

with $\beta_{mn} = \beta_{nm}$ and open boundary conditions at the edges and corners:

$$(\varepsilon - 2U)\,\beta_{11} = -2J\,\beta_{12} + P\,\beta_{22}\,, \tag{26}$$

$$\varepsilon\,\beta_{1m} = -J\,\left[\beta_{1,m-1} + \beta_{1,m+1} + \beta_{2,m}\right]\,, \tag{27}$$

$$(\varepsilon - 2U)\,\beta_{NN} = -2J\,\beta_{N,N-1}\,, \tag{28}$$

where N is the size of the system.

In this Supplementary Note, we analyze the 2D system in Fig. 2a with Kirchhoff's circuit laws and express the parameters of the Hamiltonian U and P in terms of the circuit parameters. In our analysis we assume that the voltages in the circuit nodes $U_{mn} \propto e^{-i\omega t}$, and hence $\ddot{U}_{mn} = -\omega^2\,U_{mn}$. We also assume $J = 1$ and denote

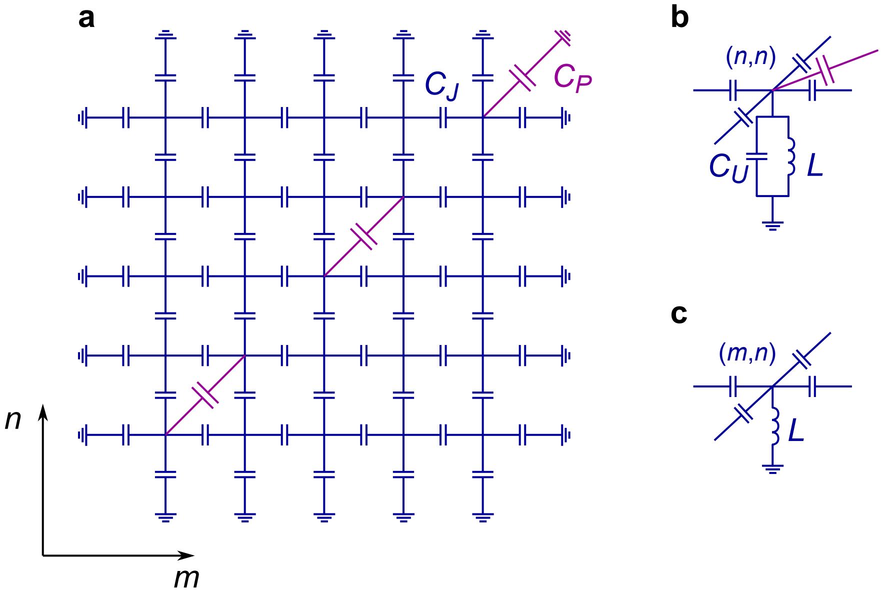

Supplementary Figure 2 a, Implementation of a 2D system emulating extended Bose-Hubbard model, top view. b, Side view of a diagonal node in the 2D system. c, Side view of an off-diagonal node in the 2D system.

$$\omega_0^2 = \frac{1}{L\,C_j}\;, \quad \varepsilon = \frac{\omega_0^2}{\omega^2} - 4\;, \tag{29}$$

$$U = \frac{C_P + C_U}{2\,C_j}\;, \quad P = -\frac{C_P}{C_j}\;. \tag{30}$$

To establish one-to-one correspondence of the initial tight-binding problem with LC circuit, we analyze several representative situations.

1. Site (m, n) with $m \neq n$, not at the edge of the system. From the first Kirchhoff's law we get

$$\frac{U_{mn}}{L} - \omega^2\,C_j\;\left[(U_{mn} - U_{m,n+1}) + (U_{mn} - U_{m,n-1}) + (U_{mn} - U_{m+1,n}) + (U_{mn} - U_{m-1,n})\right] = 0 \tag{31}$$

or with designations Supplementary Equations (29)-(30)

$$\varepsilon\,U_{mn} = -U_{m,n+1} - U_{m,n-1} - U_{m+1,n} - U_{m-1,n}\;, \tag{32}$$

which is consistent with Supplementary Equation (25).

2. Site $(1, n)$ with $n \neq 1$, at the edge of the system.

$$\frac{U_{1n}}{L} - \omega^2\,C_j\;\left[(U_{1n} - U_{1,n-1}) + (U_{1n} - U_{1,n+1}) + (U_{1n} - U_{2n}) + U_{1n}\right] = 0\;, \tag{33}$$

where the last term in the square bracket is associated with extra capacitance C_j connected to the ground. Supplementary Equation (33) can be rearranged to yield

$$\varepsilon\,U_{1n} = -U_{1,n-1} - U_{1,n+1} - U_{2,n}\;, \tag{34}$$

which reproduces open boundary condition for the tight-binding model Supplementary Equation (27). The equations for the sites $(m, 1)$, (m, N) and (N, n) (not at the corner) where N is the system size are completely analogous.

3. Site $(2m+1, 2m+1)$ *at the diagonal of the system, not the corner one.*

$$\frac{U_{2m+1,2m+1}}{L} - \omega^2\, C_j\, \left[(U_{2m+1,2m+1} - U_{2m,2m+1}) + (U_{2m+1,2m+1} - U_{2m+2,2m+1})\right.$$
$$\left. + (U_{2m+1,2m+1} - U_{2m+1,2m}) + (U_{2m+1,2m+1} - U_{2m+1,2m+2})\right] - \tag{35}$$
$$- \omega^2\, C_p\, (U_{2m+1,2m+1} - U_{2m+2,2m+2}) - \omega^2\, C_U\, U_{2m+1,2m+1} = 0\,,$$

which can be rewritten as

$$(\varepsilon - 2U)\; U_{2m+1,2m+1} = -U_{2m,2m+1} - U_{2m+2,2m+1} - U_{2m+1,2m} - U_{2m+1,2m+2} + PU_{2m+2,2m+2} \tag{36}$$

and coincides with Supplementary Equation (23) provided the pattern of voltages is symmetric, i.e. $U_{2m,2m+1} = U_{2m+1,2m}$ and $U_{2m+2,2m+1} = U_{2m+1,2m+2}$.

4. Site $(2m, 2m)$ *at the diagonal of the system*

$$\frac{U_{2m,2m}}{L} - \omega^2\, C_j\, \left[(U_{2m,2m} - U_{2m,2m+1}) + (U_{2m,2m} - U_{2m,2m-1}) +\right.$$
$$\left. + (U_{2m,2m} - U_{2m+1,2m}) + (U_{2m,2m} - U_{2m-1,2m})\right] - \tag{37}$$
$$- \omega^2\, C_p\, (U_{2m,2m} - U_{2m-1,2m-1}) - \omega^2\, C_U\, U_{2m,2m} = 0\,,$$

which can be recast in the form

$$(\varepsilon - 2U)\; U_{2m,2m} = -U_{2m,2m+1} - U_{2m,2m-1} - U_{2m+1,2m} - U_{2m-1,2m} + P\, U_{2m-1,2m-1} \tag{38}$$

in agreement with Supplementary Equation (24).

5. Site $(1, 1)$ *at the corner of the system.* For this corner site, we include two capacitances C_j to the ground to compensate the absence of the two neighbors.

$$\frac{U_{11}}{L} - \omega^2\, C_j\, \left[(U_{11} - U_{12}) + (U_{11} - U_{21}) + 2\, U_{11}\right] - \omega^2\, C_p\, (U_{11} - U_{22}) - \omega^2\, C_U\, U_{11} = 0\,, \tag{39}$$

which is equivalent to

$$(\varepsilon - 2U)\, U_{11} = -U_{12} - U_{21} + P\, U_{22} \tag{40}$$

exactly reproducing an open boundary condition Supplementary Equation (26) for the corner of the system.

6. Site (N,N) at the corner of the system (N is assumed to be odd). In contrast to $(1,1)$ site, besides two capacitances C_j connected to the ground, we also include an extra capacitance C_P connected to the ground:

$$\begin{aligned}&\frac{U_{NN}}{L}-\omega^2\,C_j\,\left[(U_{NN}-U_{N-1,N})+(U_{NN}-U_{N,N-1})+2\,U_{NN}\right]-\\&-\omega^2\,C_p\,U_{NN}-\omega^2\,C_U\,U_{NN}=0\,,\end{aligned}\tag{41}$$

which can be rearranged to yield

$$(\varepsilon-2U)\,U_{NN}=-U_{N-1,N}-U_{N,N-1}\tag{42}$$

in agreement with open boundary condition for the corner of the system, Supplementary Equation (28).

Hence, the proposed experimental setup allows us to emulate two-body physics in Bose-Hubbard model, but with several constraints on parameters that enter the Bose-Hubbard Hamiltonian: (i) P is always negative, cf. Supplementary Equation (30); (ii) $U>|P|/2$, i.e. we cannot realize the regime of too weak on-site interactions; (iii) to emulate boson states, the pattern of voltages in a circuit should be symmetric with reasonable accuracy.

Supplementary Note 4 – Analysis of topolectrical circuit with realistic losses

As we have demonstrated, the lossless 2D topolectrical circuit corresponds precisely to the Bose-Hubbard model. However, realistic circuits necessarily have losses, and in this section, we examine excitation of the system taking the effect of loss into account. We assume that the system is excited with a current source and current is pumped into one or several lattice sites. We aim to compare the governing equations with the simple driven-dissipative model outlined in Supplementary Note 2, which is described by the equation

$$\omega\,c_{mn}=\sum_{m',n'}H_{mn,m'n'}\,c_{m'n'}-i\,\gamma\,c_{mn}-i\varkappa\,E_{mn}\,,\tag{43}$$

where ω is a driving frequency, c_{mn} describe the stationary state of the system, γ is the dissipation rate and $\varkappa$ is the coupling coefficient.

In circuit analysis, we assume $e^{-i\omega t}$ time dependence of voltages, which yields the following impedances:

$$Z_L = -i\omega L + R_L \ , \quad Z_C = \frac{1}{-i\omega\, C_j} + R_C \ , \tag{44}$$

$$Z_P = \frac{1}{-i\omega\, C_P} + R_P \ , \quad Z_U = \frac{1}{-i\omega\, C_U} + R_U \ . \tag{45}$$

For sufficiently small losses, the ratios of impedances read

$$\frac{Z_C}{Z_L} = -\frac{\omega_0^2}{\omega^2} + i\left(\frac{R_L\,\omega_0^2}{L\,\omega^3} + \frac{R_C}{L\,\omega}\right) \ , \tag{46}$$

$$\frac{Z_C}{Z_P} = \frac{C_P}{C_j} + i\,\omega\,\frac{C_P}{C}\,\left(R_P\,C_P - R_C\,C_j\right) \ , \tag{47}$$

$$\frac{Z_C}{Z_U} = \frac{C_U}{C_j} + i\,\omega\,\frac{C_U}{C_j}\,\left(R_U\,C_U - R_C\,C_j\right) \ . \tag{48}$$

Similarly to Supplementary Note 3, we analyze several characteristic situations:

1. Site (m,n) with $m \neq n$, not at the edge of the system. First Kirchhoff's law now yields:

$$\frac{U_{mn}}{Z_L} + \frac{1}{Z_C}\,\left(4U_{mn} - U_{m,n-1} - U_{m,n+1} - U_{m-1,n} - U_{m+1,n}\right) = I_{mn} \tag{49}$$

which can be rearranged as

$$\varepsilon\,U_{mn} = -U_{m,n-1} - U_{m,n+1} - U_{m-1,n} - U_{m+1,n} + i\,\gamma\,U_{mn} - Z_C\,I_{mn} \ , \tag{50}$$

where γ is a frequency-dependent damping

$$\gamma = \frac{R_L\,\omega_0^2}{L\omega^3} + \frac{R_C}{L\,\omega} \ . \tag{51}$$

Note also the sign in front of γ in Supplementary Equation (51): it is different from the sign in Supplementary Equation (43). This happens due to the definition of "energy" variable Supplementary Equation (29) which ensures that imaginary parts of ω and ε have opposite signs:

$$\omega'' = -\frac{\omega_0}{2}\,\frac{\varepsilon''}{(\varepsilon' + 4)^{3/2}} \ . \tag{52}$$

Since in the dissipative case eigenmode frequency has negative imaginary part, "energy variable" ε should have positive imaginary part.

2. Site $(1,n)$ with $n \neq 1$, at the edge of the system. In a similar way we derive an equation

$$\frac{U_{1n}}{Z_L} + \frac{U_{1n}}{Z_C} + \frac{1}{Z_C}\left(3\,U_{1n} - U_{1,n-1} - U_{1,n+1} - U_{2n}\right) = I_{1n}\ , \tag{53}$$

which yields

$$\varepsilon\, U_{1n} = -U_{1,n-1} - U_{1,n+1} - U_{2n} + i\,\gamma\, U_{1n} - Z_C\, I_{1n}\ . \tag{54}$$

3. Site $(2m+1, 2m+1)$ at the diagonal of the system, not the corner one.

$$\begin{gathered}\frac{U_{2m+1,2m+1}}{Z_L} + \frac{U_{2m+1,2m+1}}{Z_U} + \frac{U_{2m+1,2m+1} - U_{2m+2,2m+2}}{Z_P} + \\ + \frac{1}{Z_C}\left(4\,U_{2m+1,2m+1} - U_{2m,2m+1} - U_{2m+2,2m+1} - U_{2m+1,2m} - U_{2m+1,2m+2}\right) = I_{2m+1,2m+1}\end{gathered} \tag{55}$$

This yields an equation

$$\begin{aligned}(\varepsilon - 2U)\, U_{2m+1,2m+1} = &-U_{2m,2m+1} - U_{2m+2,2m+1} - U_{2m+1,2m} - U_{2m+1,2m+2} + \\ &+ i\gamma'\, U_{2m+1,2m+1} + P'\, U_{2m+2,2m+2} - Z_C\, I_{2m+1,2m+1}\ ,\end{aligned} \tag{56}$$

where for the diagonal sites

$$\gamma' = \gamma + \omega\,\frac{C_P}{C_j}\left(R_P\, C_P - R_C\, C_j\right) + \omega\,\frac{C_U}{C_j}\left(R_U\, C_U - R_C\, C_j\right)\ , \tag{57}$$

$$P' = P - i\omega\,\frac{C_P}{C_j}\left(R_P\, C_P - R_C\, C_j\right)\ . \tag{58}$$

Thus, the diagonal elements have extra loss in tunneling P' and on-site losses γ' stemming from the insertion of additional elements. This problem, however, can be circumvented by requiring that

$$R_C\, C_j = R_P\, C_P = R_U\, C_U \tag{59}$$

in which case all elements (in the bulk and at the diagonal) are characterized by the same magnitude of loss, though depending on frequency. However, even if Supplementary Equation (59) is not fulfilled, doublon bands will remain almost unaffected, since the maximal voltages are expected at the diagonal sites only.

4. Site $(2m, 2m)$ at the diagonal of the system.

$$\begin{aligned}&\frac{U_{2m,2m}}{Z_L} + \frac{U_{2m,2m}}{Z_U} + \frac{U_{2m,2m} - U_{2m-1,2m-1}}{Z_P} + \\ &+ \frac{1}{Z_C}\left(4U_{2m,2m} - U_{2m-1,2m} - U_{2m+1,2m} - U_{2m,2m-1} - U_{2m,2m+1}\right) = I_{2m,2m}\end{aligned} \tag{60}$$

which yields

$$\begin{aligned}&(\varepsilon - 2U)\, U_{2m,2m} = -U_{2m-1,2m} - U_{2m+1,2m} - U_{2m,2m-1} - U_{2m,2m+1} + \\ &+ i\gamma'\, U_{2m,2m} + P'\, U_{2m-1,2m-1} - Z_C\, I_{2m,2m}\,.\end{aligned} \tag{61}$$

5. Site $(1, 1)$ at the corner of the system.

$$\left(Z_L^{-1} + Z_U^{-1} + 2\, Z_C^{-1}\right) U_{11} + \frac{1}{Z_C}\left(2U_{11} - U_{12} - U_{21}\right) + \frac{1}{Z_P}\left(U_{11} - U_{22}\right) = I_{11}\,, \tag{62}$$

which yields

$$(\varepsilon - 2U)\, U_{11} = i\gamma'\, U_{11} - U_{12} - U_{21} + P'\, U_{22} - Z_C\, I_{11}\,. \tag{63}$$

6. Site (N, N) at the corner of the system [N is assumed to be odd].

$$\left(Z_L^{-1} + Z_U^{-1} + Z_P^{-1} + 2\, Z_C^{-1}\right) U_{NN} + \frac{1}{Z_C}\left(2\, U_{NN} - U_{N-1,N} - U_{N,N-1}\right) = I_{NN} \tag{64}$$

which can be transformed as

$$(\varepsilon - 2U)\, U_{NN} = i\,\gamma'\, U_{NN} - U_{N-1,N} - U_{N,N-1} - Z_C\, I_{NN}\,. \tag{65}$$

To summarize, the designed LC circuit is described by the same driven-dissipative system Supplementary Equations (43) apart from the following differences:

- dissipation coefficient γ depends on the frequency of driving ω;
- the magnitude of dissipation for diagonal and off-diagonal sites is different unless an additional constraint Supplementary Equation (59) is fulfilled;

- instead of frequency in Supplementary Equation (43), we deal with the auxiliary "energy variable" ε which is inversely proportional to frequency and therefore has a positive imaginary part in the dissipative case.
- instead of external field E_{mn} we have a combination $Z_C I_{mn}$ which is frequency-dependent.

In all other aspects, the standard driven-dissipative model captures all essential features of the proposed topolectrical circuit and hence the tomography technique developed in Supplementary Note 2 can be applied to the designed LC circuit.

Supplementary Note 5 – Distribution of elements in the experimental setup

In contrast to the idealized model of electric circuit discussed above, the actual values of inductances and capacitances of setup elements slightly fluctuate from one element to another. Hence, the experimental circuit possesses an inherent disorder in the tunneling constants J and P (ascribed to capacitors C_J and C_P) as well as in the interaction strength U and on-site resonant frequencies ω_0, depending on capacitors C_U and inductors L, respectively. The elements used in our experimental sample are Murata GRM32RR71H105KA01 for C_J, Murata GRM31CR61A476ME15L for C_P, Murata GRM31CR71C106KAC7 for C_U, and Bourns RLB1314-220KL for L. In the process of fabrication, we created a detailed map of elements that allows us to determine the precise value of the given circuit bond. The distributions of the element values are shown in Supplementary Figure 3. As seen from the histograms, actual mean values in the fabricated circuit are $L = 22.77\,\mu$H, $C_J = 1.0024\,\mu$F, $C_U = 10.031\,\mu$F, and $C_P = 4.1863\,\mu$F which are slightly different from the ones provided in specifications. Typical fluctuations of parameters are of the order of 1-2%. We use these measured mean values for numerical simulations described in the Article main text.

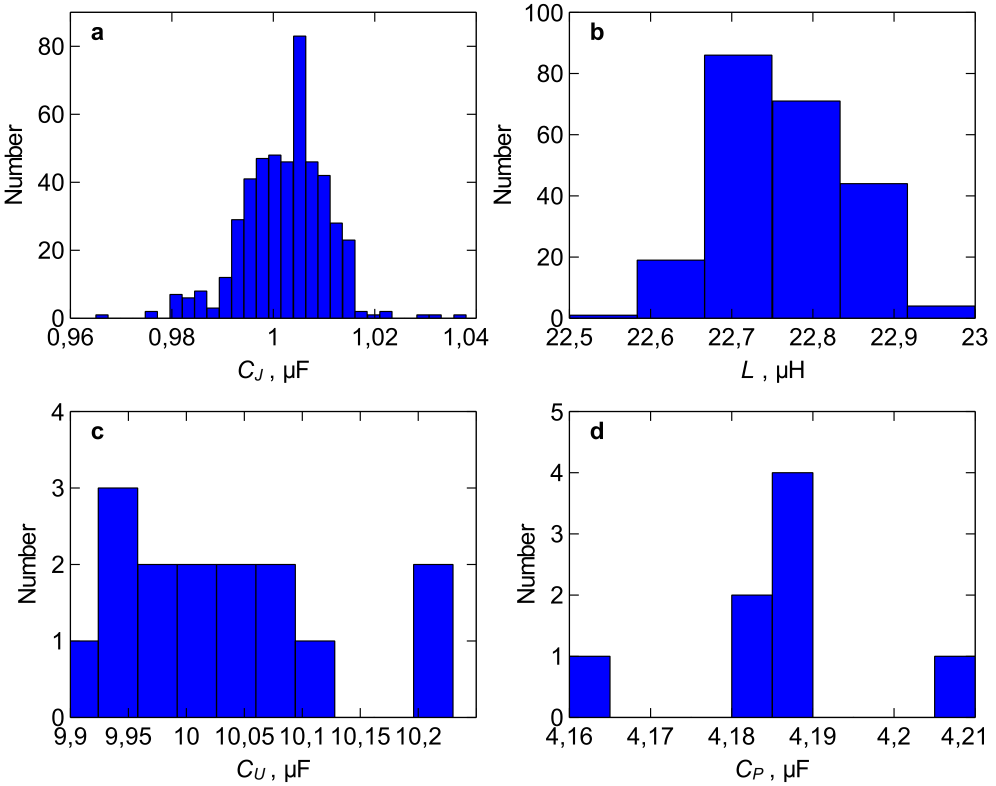

Supplementary Figure 3 Distribution of the lumped element values in the experimental setup for (a) capacitors C_J, (b) inductors L, (c) C_U and (d) C_P. Different number of bins in panels (a) and (b) is due to the different precision of measurements in cases (a-d). Total amounts of corresponding elements in the circuit are 225 for L, 480 for C_J, 15 for C_U, and 8 for C_P.

Supplementary Note 6 – Mutual inductance

Some elements in the experimental setup can have extra parasitic couplings besides the couplings introduced intentionally. This effect is especially pronounced for inductive coils, which have relatively large size compared to the inter-coil distance, as can be seen from Fig. 1 of the article main text. We estimate now the effects of such inductive coupling on the performance of the studied setup.

Incorporating mutual inductance between the coils (grounding elements) into the Kirchhoff's rule for

the site $(2m+1, 2m+1)$, which we consider as an example, we get:

$$
\begin{aligned}
&(U_{2m+1,2m+1} + U_{\text{ind}})(Z_L^{-1} + Z_U^{-1}) + (U_{2m+1,2m+1} - U_{2m+2,2m+2})Z_P^{-1} + \\
&+ (4U_{2m+1,2m+1} - U_{2m,2m+1} - U_{2m+2,2m+1} - U_{2m+1,2m} - U_{2m+1,2m+2})Z_C^{-1} = \\
&= I_{2m+1,2m+1},
\end{aligned}
\tag{66}
$$

where U_{ind} denotes the voltage induced in the coil connecting the site $(2m+1, 2m+1)$ to the ground by all surrounding coils. A diagonal capacitor C_P is present between the sites $(2m+1, 2m+1)$ and $(2m+2, 2m+2)$. Taking into account the interaction of the given coil with its nearest neighbors and also with the coils having both coordinates m, n shifted by 1 (diagonal neighbors), we can express this induced voltage as

$$
\begin{aligned}
U_{\text{ind}} = &-Z_M(I^{(g)}_{2m,2m+1} + I^{(g)}_{2m+2,2m+1} + I^{(g)}_{2m+1,2m} + I^{(g)}_{2m+1,2m+2}) - \\
&- Z'_M(I^{(g)}_{2m,2m} + I^{(g)}_{2m,2m+2} + I^{(g)}_{2m+2,2m} + I^{(g)}_{2m+2,2m+2}),
\end{aligned}
\tag{67}
$$

where $I^{(g)}_{m,n}$ is a current through the inductance which connects the site (m, n) to the ground, Z_M and Z'_M are the impedances corresponding to the mutual inductance between the nearest neighbors and diagonal neighbors, respectively.

In the limit of strong interaction $U \gg 1$, which is the case in the considered model, all voltages are mostly concentrated at the diagonal sites of the circuit in the frequency range of interest. Then, the above expression takes the form

$$
U_{\text{ind}} \approx -Z'_M\Big(\frac{U_{2m+1,2m+1} - U_{2m+2,2m+2}}{Z_P} + I_{2m+2,2m+2}\Big) - Z'_M I_{2m,2m},
\tag{68}
$$

where $I_{2m,2m}$ and $I_{2m+2,2m+2}$ denote external currents applied to the corresponding sites. Then, Supplementary Equation (66) reads

$$
\begin{aligned}
&U_{2m+1,2m+1}(Z_L^{-1} + Z_U^{-1})\Big(1 - \frac{Z'_M}{Z_P}\Big) + (U_{2m+1,2m+1} - U_{2m+2,2m+2})Z_P^{-1} = \\
&= I_{2m+1,2m+1} + Z'_M(Z_L^{-1} + Z_U^{-1})(I_{2m,2m} + I_{2m+2,2m+2}).
\end{aligned}
\tag{69}
$$

If only one site of the circuit is driven, which is the case in our measurements, then the second term at the right-hand side vanishes, and we finally obtain the equation

$$U_{2m+1,2m+1}(Z_L^{-1}+Z_U^{-1})\left(1-\frac{Z'_M}{Z_P}\right)+(U_{2m+1,2m+1}-U_{2m+2,2m+2})Z_P^{-1}=I_{2m+1,2m+1}, \tag{70}$$

which has the same form as the equation describing the circuit without inductive couplings up to renormalization of the effective model parameters caused by the extra factor $(1-Z'_M/Z_P)$.

Supplementary Note 7 – Circuit impedance and doublon spectroscopy

To demonstrate doublon states experimentally, we need a technique to distinguish doublon modes from the rest of the modes supported by the two-dimensional sample. In this regard, it is useful to consider the quantity [2]

$$S_m(f)=\sum_n |\varphi_{nn}^{mm}|^2, \tag{71}$$

where φ_{ij}^{mn} is the potential at the site (i,j) of the circuit when the external driving voltage at frequency f is applied to the site (m,m). Since doublon modes are characterized by the voltage maxima at the diagonal sites, this quantity exhibits resonant peaks at frequencies of doublon modes. If the external voltage is applied to the site (N,N), where $N=15$ in our case, a single peak corresponding to the doublon edge state is observed. On the other hand, if any other diagonal site is driven, then two peaks centered at frequencies of bulk doublon bands emerge, Supplementary Figure 4a.

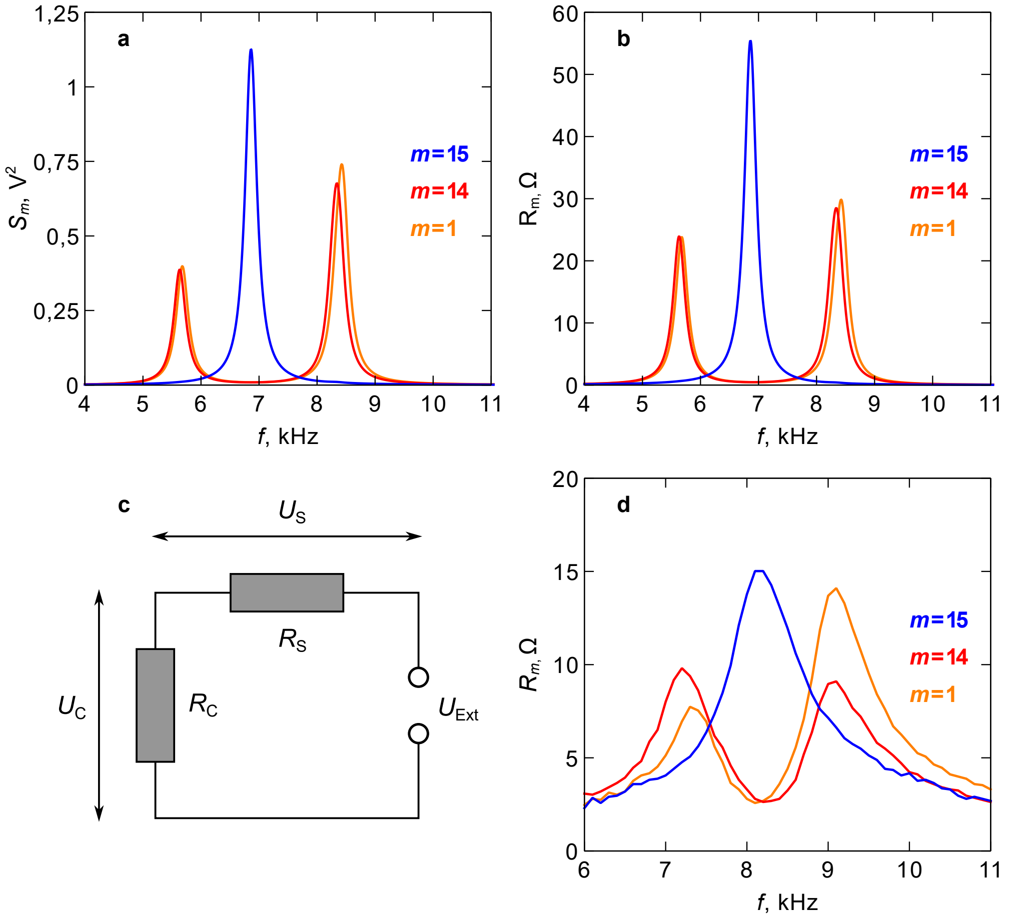

Supplementary Figure 4 (a) Numerically simulated doublon spectroscopy relying on the protocol with quantity S_m, Supplementary Equation (71), for $m = 1{,}14$ and 15. (b) Circuit impedance spectroscopy showing the real part R_m of the total impedance between the given site (m, m) and ground. Both panels are calculated for the values of circuit elements taken from the exact map of the experimental setup. (c) Equivalent scheme for the experimental setup including an external voltage source $U_{\rm ext}$ with the resistance R_s and the associated voltage drop U_s. R_c denotes the real part of the total impedance between the driven site of the circuit and the ground with the associated voltage drop U_c. (d) Experimental results of the circuit impedance spectroscopy.

We now demonstrate that the frequency dependence of the quantity S_m is consistent with the results

of the circuit impedance spectroscopy. Indeed, one can introduce Green's matrix of the circuit $\hat{G}$ [3], which is by definition related to the admittance matrix $\hat{Y}$ introduced in the main text as

$$G_{mn,m'n'} = (Y^{-1})_{mn,m'n'} \tag{72}$$

Then, potentials at the nodes of the externally driven circuit are related to the driving current I_{mn} as

$$\varphi_{mn} = \sum_{m',n'} G_{mn,m'n'} \, I_{m'n'}, \tag{73}$$

and therefore $\varphi_{nn}^{mm} = G_{nn,mm} \, I_{\text{ext}}$ where I_{ext} is a value of the driving current. Hence, Supplementary Equation (71) takes the form

$$S_m = I_{\text{ext}}^2 \sum_n |G_{nn,mm}|^2 \,. \tag{74}$$

At the same time, the characteristic impedance R_m between the given node (m, m) of the circuit and the ground is simply given by the diagonal element of the Green's matrix:

$$R_m = \text{Re}\{G_{mm,mm}\}. \tag{75}$$

As seen from Supplementary Figure 4b, the characteristic impedance of the circuit R_m demonstrates a very similar structure of resonant peaks compared to the quantity S_m for various positions of the driven site (m, m), which highlights the dominant role of diagonal entries of the Green's matrix in our system.

The equivalent scheme of the experimental setup with an external source applied to the diagonal site has the form shown in Supplementary Figure 4c. It represents a series connection of the total circuit impedance with the real part R_c and the voltage source equivalent impedance $R_s = 50\,\Omega$. In the experiment, the voltage drop between the given site of the circuit and the ground U_c is measured as a function of the driving frequency f. The external voltage U_{ext} is fixed and set to the value 1 V. Then, the circuit impedance R_c can be obtained as

$$R_c(f) = \frac{U_c(f)}{U_{\text{ext}} - U_c(f)} R_s, \tag{76}$$

while the external current flowing into the circuit is given by the relation $I_{\text{ext}}(f) = U_{\text{ext}}/(R_c(f) + R_s)$. All calculations in the article main text are carried on for the constant external current I_{ext} flowing into the system, whereas the experimental data is obtained for the fixed external voltage U_{ext}. To make a fair comparison of these results, we multiply the experimental values of $S_m(f)$ by the factor $I^2(f_0)/I^2(f)$ with $f_0 = 6$ kHz defining a calibrating value of the external current. Experimental results on doublon spectroscopy recalculated in this way are presented in Fig. 2c of the article main text.

Supplementary Note 8 – Scattering states

Besides two bulk doublon bands and doublon edge state, there exists a vast set of the system eigenmodes termed as scattering states. These modes shown by the shaded region in the dispersion (Fig. 2a of the article main text) correspond to such a state of two photons when they are typically located at distinct resonators and possess energy equal to the sum of single-photon energies. Supplementary Figure 5 shows the probability distributions $|\beta_{mn}|^2$ for such states obtained by the diagonalization of the tight-binding Hamiltonian in the absence of dissipation. Here, panels (a-d) correspond to the eigenmodes of an ideal system without any disorder. Introducing the disorder in coupling constants J and P with the uniform distribution within the range $\pm 10\%$, we observe only slight changes in the eigenmode intensity patterns accompanied by weak energy shifts, Supplementary Figure 5(e-h). However, when the strength of disorder is increased further up to 30%, quite strong distortions of eigenmode profiles are observed and the energy shifts become comparable with the spectral distance between the spectrally close modes, Supplementary Figure 5(i-l).

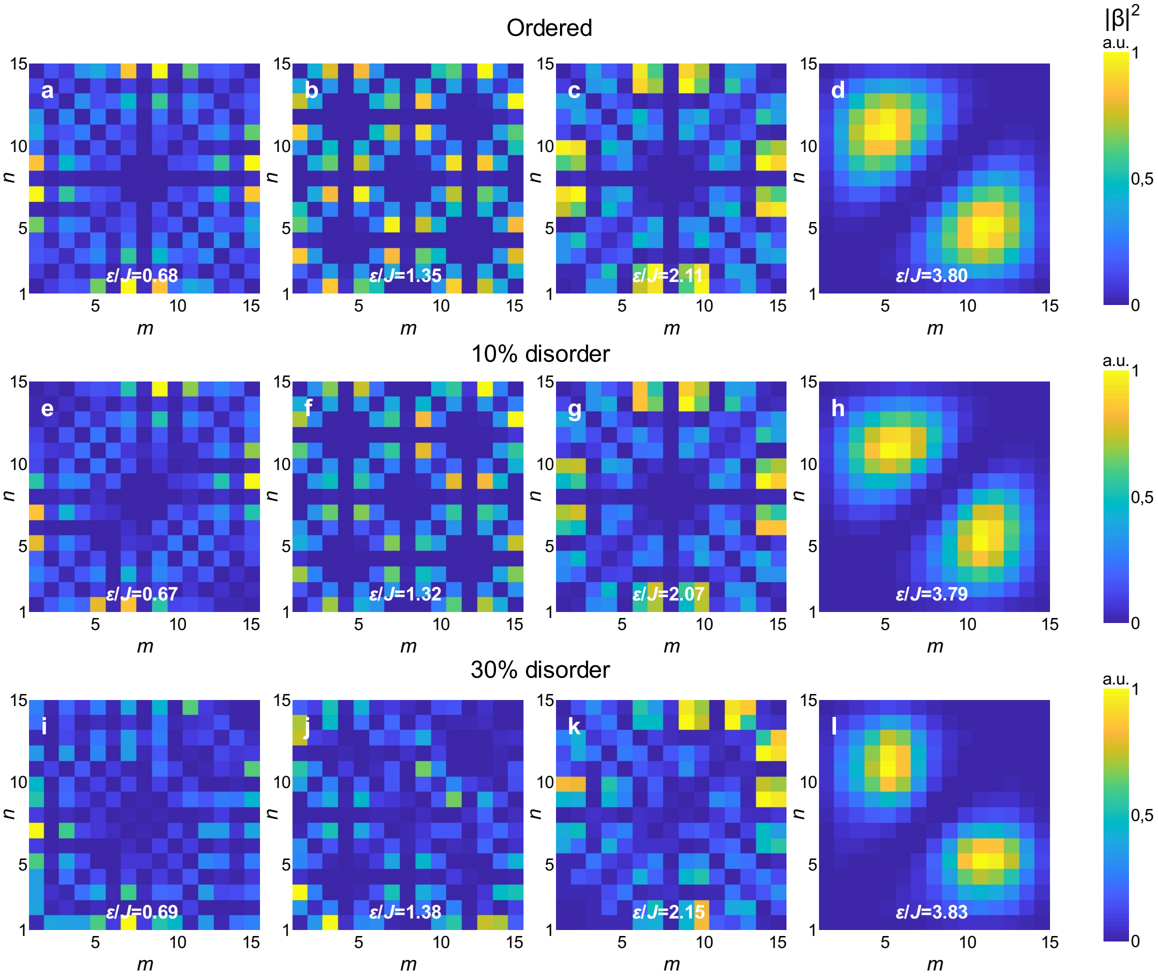

Supplementary Figure 5 Scattering states of photon pair with energies (a) $\varepsilon/J = 0.68$, (b) $\varepsilon/J = 1.35$, (c) $\varepsilon/J = 2.11$, and (d) $\varepsilon/J = 3.80$. Color encodes the magnitude of the two-photon probability distribution $|\beta_{mn}|^2$. Panels (a)-(d) are obtained by diagonalization of the tight binding Hamiltonian for the model without disorder. (e-h) Scattering states corresponding to the same eigenmodes as in the top row, but for a uniform $\pm 10\%$ disorder in the strength of bonds. (i-l) The same modes as in (e-h), but for a $\pm 30\%$ bond disorder.

Supplementary Note 9 – Effects of disorder

To examine the impact of disorder and losses on the results of tomography procedure (Supplementary Note 2), we simulate eigenmode tomography for the designed circuit taking into account Ohmic losses as well as fluctuations in the values of all lumped elements L, C_J, C_U, and C_P. For the sake of simplicity, we consider uniformly distributed fluctuations, and Ohmic losses are assumed to be constant and equal to their maximal possible values within the considered spectral range according to elements specifications.

The evolution of low-energy doublon mode, doublon edge state, and high-energy doublon mode with the increase of disorder is shown in panels (a-c), (d-f), and (g-i) of Supplementary Figure 6, respectively. It is seen that enhanced localization of doublon modes can be observed even at 10% disorder, which finally results in the formation of localized states in the bulk of the circuit at 30% disorder. At the same time, the edge state easily survives 10% disorder, since it is spectrally well-separated from the bulk doublon bands having a relatively small spatial overlap with them. However, strong 30% disorder can mix it with bulk doublon states, as seen in Supplementary Figure 6f. It should be stressed that the considered levels of disorder in the values of circuit elements exceed those expected for the experimental circuit and the corresponding results are calculated for the illustrative purpose only.

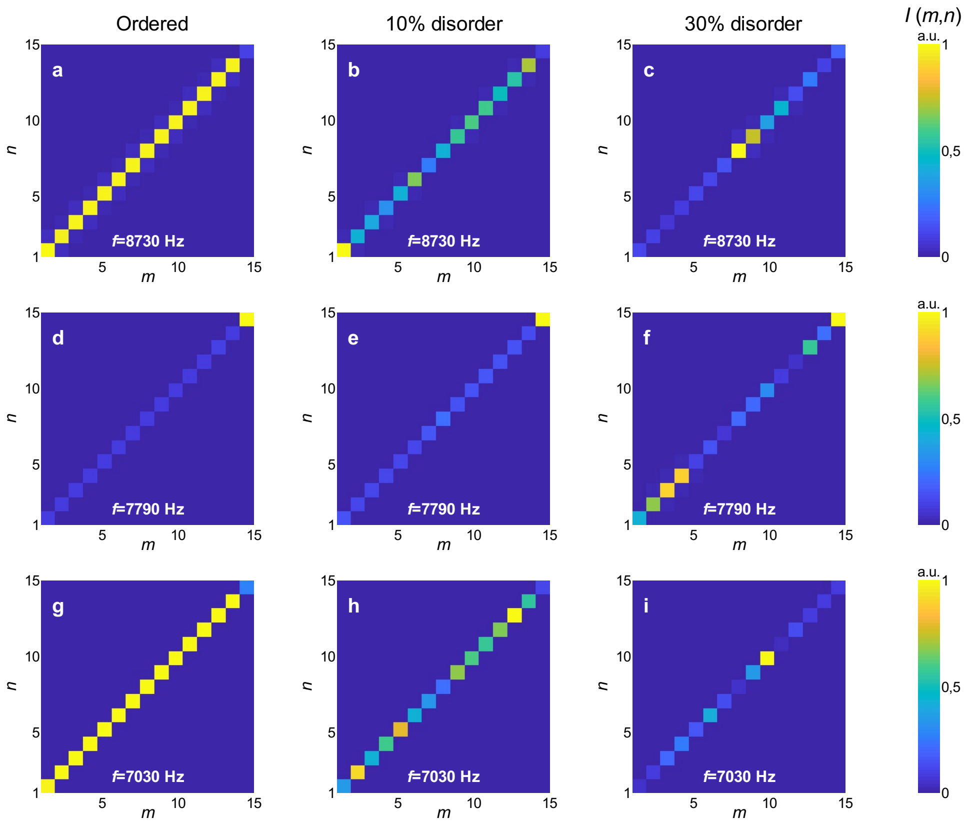

Supplementary Figure 6 Simulation of eigenmode tomography in circuits with various levels of disorder in element values. (a-c) Low-energy (high-frequency) doublon mode, $f = 8730$ Hz. (d-f) doublon edge state, $f = 7790$ Hz. (g-i): high-energy (low-frequency) doublon mode, $f = 7030$ Hz. Simulation of tomography is performed at frequencies corresponding to the peak positions in doublon spectroscopy simulation.

Supplementary Note 10 – Evaluation of topological invariant from experimental data

The definition of topological invariant for interacting many-body systems is currently an open problem which is being actively investigated. In our specific case, however, the clue to topological characterization is provided by the fact that the effective doublon Hamiltonian corresponds to that of the Su-Schrieffer-Heeger model once strong interaction regime $U \gg J$ is realized. This is the case for our experimental sample with

$U/J = 7.09$ and $P/J = -4.18$.

The effective Hamiltonian for doublons written in the basis of isolated resonators' eigenstates takes the form (see Supplementary Note 1 for details):

$$\hat{H}(k) = \begin{pmatrix} 0 & j_1 + j_2\, e^{-ik} \\ j_1 + j_2\, e^{ik} & 0 \end{pmatrix} , \tag{77}$$

where $j_1 = J^2/U$ and $j_2 = j_1 + P$ are effective tunneling amplitudes for doublons. Hence, for the given Bloch eigenmode the ratio of two components of the doublon wave function $|\psi\rangle = (\psi_{\rm A}, \psi_{\rm B})^T$ is given by:

$$\frac{\psi_{\rm A}}{\psi_{\rm B}} = \frac{j_1 + j_2\, e^{-ik}}{\varepsilon(k)} . \tag{78}$$

We notice that the effective doublon Hamiltonian Supplementary Equation (77) is represented in chiral basis and therefore the winding number is determined by plotting its off-diagonal block $q(k) = j_1 + j_2\, e^{-ik}$ on the complex plane [4]. Furthermore, according to Supplementary Equation (78) $q(k) = \varepsilon(k)\, \psi_{\rm A}/\psi_{\rm B}$, where doublon energy $\varepsilon(k)$ is purely real. Hence, the winding number can be found by plotting the ratio $\psi_{\rm A}/\psi_{\rm B}$ for a particular Bloch eigenmode.

In an experimental situation, we measure the distribution of voltages at the diagonal of the sample: $U_{\rm A}(n)$ and $U_{\rm B}(n)$. To get a result, corresponding to the given value of wave number k, we perform a discrete Fourier transform of those voltages extracting

$$U_{\rm A}(k) = \sum_n U_{\rm A}(n) e^{-ikn} , \tag{79}$$

$$U_{\rm B}(k) = \sum_n U_{\rm B}(n) e^{-ik(n-1)} \tag{80}$$

and then plotting the ratio $U_{\rm A}(k)/U_{\rm B}(k)$ on the complex plane.

Calculating the winding number from experimental data, we assume that the pattern of voltages excited in a topolectrical circuit by the source inserted in the middle of the diagonal resembles the pattern of

voltages expected for the eigenmode, which is justified in the case of doublon bands well-separated from the scattering continuum.

Supplementary References

1. Su, W. P., Schrieffer, J. R. & Heeger, A. J. Solitons in Polyacetylene. *Phys. Rev. Lett.* **42**, 1698–1701 (1979).

2. Gorlach, M. A. *et al.* Simulation of two-boson bound states using arrays of driven-dissipative coupled linear optical resonators. *Phys. Rev. A* **98**, 063625 (2018).

3. Chung, F. & Yau, S.-T. Discrete Green's Functions. *Journal of Combinatorial Theory, Series A* **91**, 191–214 (2000).

4. Ryu, S., Schnyder, A. P., Furusaki, A. & Ludwig, A. W. W. Topological insulators and superconductors: tenfold way and dimensional hierarchy. *New J. Phys.* **12**, 065010 (2010).
